# Prevalence of Discrimination and the Association Between Employment Discrimination and Health Care Access and Use **—** National HIV Behavioral Surveillance Among Transgender Women, Seven Urban Areas, United States, 2019***–***2020

**DOI:** 10.15585/mmwr.su7301a6

**Published:** 2024-01-25

**Authors:** Amy R. Baugher, Evelyn Olansky, Larshie Sutter, Susan Cha, Rashunda Lewis, Elana Morris, Christine Agnew-Brune, Lindsay Trujillo, Ebony Respress, Kathryn Lee, National HIV Behavioral Surveillance Among Transgender Women Study Group, National HIV Behavioral Surveillance Among Transgender Women Study Group, Narquis Barak, Kathleen A. Brady, Sarah Braunstein, Jasmine Davis, Sara Glick, Andrea Harrington, Jasmine Lopez, Yingbo Ma, Aleks Martin, Genetha Mustaafaa, Tanner Nassau, Gia Olaes, Jennifer Reuer, Alexis Rivera, William T. Robinson, Ekow Kwa Sey, Sofia Sicro, Brittany Taylor, Dillon Trujillo, Erin Wilson, Pascale Wortley

**Affiliations:** ^1^Division of HIV Prevention, National Center for HIV, Viral Hepatitis, STD, and TB Prevention, CDC, Atlanta, Georgia; ^2^Social & Scientific Systems, Inc., Silver Spring, Maryland; ^3^ICF, Fairfax, Virginia; ^4^Oak Ridge Institute for Science and Education, Oak Ridge, Tennessee; CrescentCare; Philadelphia Department of Public Health; New York City Department of Health and Mental Hygiene; CrescentCare; University of Washington, School of Medicine, Division of Allergy and Infectious Diseases, Public Health – Seattle & King County, HIV/STD Program; Philadelphia Department of Public Health; New York City Department of Health and Mental Hygiene; Los Angeles County Department of Public Health; , Public Health – Seattle & King County, HIV/STD Program; Georgia Department of Public Health; Philadelphia Department of Public Health; Los Angeles County Department of Public Health; Washington State Department of Health; New York City Department of Health and Mental Hygiene; Louisiana State University Health Science Center in New Orleans – School of Public Health, Louisiana Office of Public Health STD/HIV/Hepatitis Program; Los Angeles County Department of Public Health; San Francisco Department of Public Health; Georgia Department of Public Health; San Francisco Department of Public Health; San Francisco Department of Public Health; Georgia Department of Public Health.

## Abstract

Transgender women experience discrimination in many settings, including in employment. Because employment and health insurance are intertwined in the United States, employment discrimination might be related to lower health insurance coverage and health care use, including gender-affirming care. This analysis used data from transgender women (N = 1,608) in seven urban areas in the United States collected during 2019–2020 to present the prevalence of six discrimination types (employment, housing, bathroom, businesses, health care, and abuse) and to measure the association between employment discrimination (defined as trouble getting a job or fired due to being transgender) and sociodemographic characteristics, health care access, and health care use. Log-linked Poisson regression models were conducted to estimate adjusted prevalence ratios and 95% CIs. Seven in 10 transgender women experienced at least one type of discrimination during the past 12 months. During the same period, 9.9% of transgender women were fired and 32.4% had trouble getting a job because of being transgender. Employment discrimination was associated with younger age and lower socioeconomic status. Having trouble getting a job was associated with health care access and health care use factors, including having no health insurance or having Medicaid only, having an unmet medical need because of cost, never having transgender-specific care, and having an unmet need for gender-affirming procedures. These findings suggest that employment discrimination contributes to transgender women’s economic marginalization and their ability to obtain adequate health insurance coverage and achieve their transition goals. These findings might help guide efforts that protect transgender women’s right to pursue their work, health, and life goals without discrimination.

## Introduction

Transgender women have historically been marginalized in public spaces and institutions, including the workplace (*1*). In the United States, discrimination against job applicants or employees by employers on the basis of gender identity or transgender status is illegal (*2*), yet discrimination persists (*3*,*4*). Employment discrimination operates as a multilevel phenomenon (*5*–*7*): structural (e.g., law), organizational (e.g., workplace policies regarding identification and legal names), interpersonal (e.g., inappropriate questions from coworkers), and individual (e.g., health and financial outcomes). Because employment and health insurance are intertwined in the United States, employment discrimination might be related to lower health insurance coverage and care use (*8*), including gender-affirming care, which is important for transgender women’s mental health (*9*), quality of life (*10*), transition goals (*11*), and HIV prevention and care engagement (*12*). In addition to employment discrimination, discrimination of any type is related to delays in health care (*13*,*14*), suicidal ideation (*15*), and negative health outcomes (*1*) among transgender women. Therefore, it is important to understand the prevalence of multiple types of discrimination that transgender women experience. Previous reports on discrimination among transgender women focus on transgender women who are predominantly White and have higher socioeconomic status (SES) (*3*); this analysis was conducted to understand discrimination in a diverse and lower SES population.

The objectives of this analysis were to describe the prevalence of multiple types of discrimination toward transgender women and to measure the characteristics of employment discrimination and its association with health care access and use. Policymakers can use these results to guide civil rights legislation efforts.

## Methods

### Data Source

This report includes survey data from the National HIV Behavioral Surveillance Among Transgender Women (NHBS-Trans) conducted by CDC during June 2019–February 2020 to assess health and prevention behaviors and HIV prevalence (*16*). Eligible participants completed an interviewer-administered questionnaire and were offered HIV testing. Additional information about NHBS-Trans eligibility criteria, data collection, and biologic testing is available in the overview and methodology report of this supplement (*17*). The NHBS-Trans protocol, questionnaire, and documentation are available at https://www.cdc.gov/hiv/statistics/systems/nhbs/methods-questionnaires.html#trans.

Applicable local institutional review boards in each participating project area approved NHBS-Trans activities. The final NHBS-Trans sample included 1,608 transgender women in seven urban areas in the United States (Atlanta, Georgia; Los Angeles, California; New Orleans, Louisiana; New York, New York; Philadelphia, Pennsylvania; San Francisco, California; and Seattle, Washington) recruited using respondent-driven sampling. This activity was reviewed by CDC, deemed not research, and was conducted consistent with applicable Federal law and CDC policy.^*^

### Measures

Six measures for discrimination types were assessed: 1) employment (fired or had trouble getting a job), 2) housing (denied housing or evicted), 3) bathroom (denied bathroom access), 4) discrimination in businesses (treated poorly in businesses), 5) health care (denied or given lower-quality health care), and 6) abuse (verbally abused or physically abused). Other measures included health outcomes, health care access and use, and gender-affirming care.

Demographics and social determinants of health were measured, including age, race and ethnicity, poverty, homelessness, severe food insecurity, incarceration, disability, and sex work. Definitions of discrimination, demographics, and social determinants of health are available in the overview report of this supplement (*17*). Health care access variables included currently having health insurance, type of health insurance, living in a state where Medicaid laws explicitly covered gender-affirming care in 2019 when data were collected (*18*), having a usual source of care, unmet need for health care because of cost, health insurance coverage for hormone therapy among transgender women with health insurance, and transgender-specific health care. Health care use included visiting any health care provider during the past 12 months, unmet need for hormone therapy, using nonprescription hormones among transgender women who used any hormones, and unmet need for gender-affirmation procedures (Table 1).

**TABLE 1:** Measures, questions, and analytic coding for prevalence of discrimination and the association between employment discrimination and health care access and use, by type of discrimination and selected characteristics — National HIV Behavioral Surveillance Among Transgender Women, seven urban areas,* United States, 2019–2020

Measure	Question	Analytic coding
**Discrimination type**		
Employment discrimination	In the past 12 months, have you been fired from a job because you are transgender or gender nonconforming? Had trouble getting a job because you are transgender or gender nonconforming?	Yes or no
Bathroom discrimination past 12 months	In the past 12 months, have you been denied access to bathrooms that were appropriate to your gender identity?	Yes or no
Housing discrimination past 12 months	In the past 12 months, have you been denied housing or been evicted because you are transgender or gender nonconforming?	Yes or no
Health care discrimination past 12 months	In the past 12 months, have you been denied or given lower quality health care because you are transgender or gender nonconforming?	Yes or no
Discrimination in businesses	In the past 12 months, have you received poorer services than other people in restaurants, stores, or businesses because you are transgender or gender nonconforming?	Yes or no
Abuse	In the past 12 months, have you been verbally abused or harassed because of your gender identity or presentation? Been physically abused or harassed because of your gender identity or presentation?	Yes or no
**Health outcome**		
HIV status	NHBS biologic HIV test result	Negative, positive, unknown result, or did not consent to test
Disability^†^	Are you deaf or do you have serious difficulty hearing? Are you blind or have serious difficulty seeing, even when wearing glasses? Because of a physical, mental, or emotional condition, do you have serious difficulty concentrating, remembering, or making decisions? Do you have serious difficulty walking or climbing stairs? Do you have difficulty dressing or bathing? Because of a physical, mental, or emotional condition, do you have difficulty doing errands alone, such as visiting a doctor’s office or shopping?	Yes or no
**Health care access and use**		
State Medicaid laws explicitly cover gender-affirming care, 2019^§^	City of residence	Yes or no
Usual source of health care	Is there a place that you usually go when you are sick or you need advice about your health? Please do not include Internet websites.	Yes or no
Visited health care provider past 12 months	In the past 12 months, that is, since [fill with interview month, formatted as text] of last year, have you seen a doctor, nurse, or other health care provider?	Yes or no
Unmet need for health care because of cost past 12 months	During the past 12 months, was there any time when you needed medical care but didn’t get it because you couldn’t afford it?	Yes or no
Comfort with health care provider	Do you have a health care provider with whom you feel comfortable discussing gender-related health issues?	Yes or no
**Gender-affirming care**		
Unmet need for hormone therapy	Have you ever taken hormones for gender transition or affirmation? Are you currently taking hormones for gender transition or affirmation? Would you like to take hormones for gender transition or affirmation?	Yes or no
Health insurance covers hormone therapy	Does your current health insurance cover hormones for gender transition or affirmation?	Yes or no
Used nonprescription hormones past 12 months	In the past 12 months, have you used hormones that were not prescribed to you by a doctor or other health care professional?	Yes or no
Unmet need for gender-affirmation procedure	Have you ever had any type of surgery for gender transition or affirmation? Do you plan or want to get additional surgeries for gender transition or affirmation? Do you want to have surgery for gender transition or affirmation?	Yes or no

**Abbreviation:** NHBS = National HIV Behavioral Surveillance.

* Atlanta, GA; Los Angeles, CA; New Orleans, LA; New York City, NY; Philadelphia, PA; San Francisco, CA; and Seattle, WA.

^†^ To assess difficulty in six basic domains of functioning (hearing, vision, cognition, walking, self-care, and independent living), based on U.S. Department of Health and Human Services disability data standard (https://aspe.hhs.gov/reports/hhs-implementation-guidance-data-collection-standards-race-ethnicity-sex-primary-language-disability-0).

^§^ State Medicaid coverage as of 2019 was determined by the Williams Institute’s October 2019 report (https://williamsinstitute.law.ucla.edu/wp-content/uploads/Medicaid-Gender-Care-Oct-2019.pdf).

### Analysis

Log-linked Poisson regression models with generalized estimating equations clustered on recruitment chain were used to obtain adjusted prevalence ratios and 95% CIs. Referent groups were selected based on who was expected to have the most favorable outcome. Models comparing group differences in employment discrimination were adjusted for urban area and network size (*19*). Models comparing trouble getting a job to health care access and use outcomes were adjusted for urban area, network size, and age. Certain categories were not modeled because of sparse data. Statistical significance was determined by whether the CI crossed the null of 1.0. Analyses were conducted using SAS software (version 9.4; SAS Institute).

## Results

Overall, 69.9% of 1,608 transgender women in seven urban areas experienced at least one type of discrimination during the past 12 months because of being transgender. Among transgender women, 53.9% were verbally abused; 39.1% received poorer service in restaurants, stores, or businesses; 32.4% had trouble getting a job; 26.6% were physically abused; 22.3% were denied access to a gender-affirming bathroom; 13.9% were denied housing or evicted; 10.8% were denied or given lower quality health care; and 9.9% were fired from a job (Figure).

**FIGURE:**
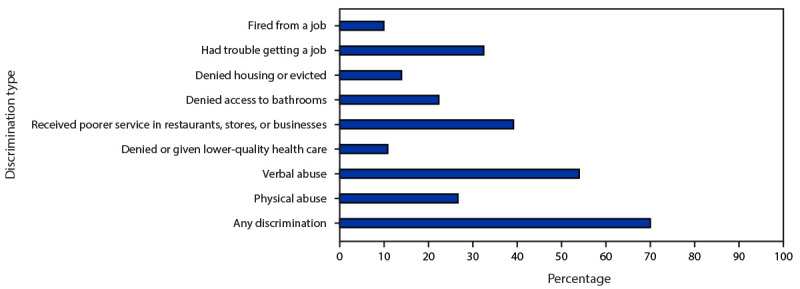
Prevalence of types of transgender-specific discrimination during the past 12 months among transgender women — National HIV Behavioral Surveillance Among Transgender Women, seven urban areas,* United States, 2019–2021^†^ * Atlanta, GA; Los Angeles, CA; New Orleans, LA; New York City, NY; Philadelphia, PA; San Francisco, CA; and Seattle, WA. ^†^ N = 1,608 participants.

Transgender women aged 18–29 years were more likely to be fired because of being transgender than those who were aged ≥50 years (Table 2). Transgender women who reported experiencing homelessness and severe food insecurity were more likely to have been fired during the past year because of being transgender than those who did not have those experiences.

**TABLE 2:** Number and percentage of transgender women experiencing transgender-specific employment discrimination during the past 12 months, by selected characteristics — National HIV Behavioral Surveillance Among Transgender Women, seven urban areas,* United States, 2019–2020^†^

Characteristic	Total no.	Fired from a job (n = 158)		Trouble getting a job (n = 513)	
		No. (%)^§^	aPR^¶^ (95% CI)	No. (%)^§^	aPR^¶^ (95% CI)
**Age group, yrs**					
18–29	**496**	64 (12.9)	1.71 (1.08–2.71)	214 (43.1)	2.47 (1.97–3.09)**
30–39	**461**	45 (9.7)	1.36 (0.76–2.42)	149 (32.3)	1.93 (1.53–2.43)**
40–49	**307**	26 (8.5)	1.22 (0.75–1.96)	92 (30.0)	1.71 (1.42–2.06)**
≥50	**343**	23 (6.7)	Ref	58 (16.9)	Ref
**Race and ethnicity^††^**					
American Indian or Alaska Native	**17**	2 (11.8)	—^§§^	5 (29.4)	—
Asian	**30**	3 (10.0)	—	4 (13.3)	—
Black or African American	**569**	54 (9.5)	1.07 (0.68–1.69)	125 (22.0)	0.54 (0.40–0.72)**
Native Hawaiian or other Pacific Islander	**42**	1 (2.4)	—	6 (14.3)	—
White	**180**	19 (10.6)	Ref	79 (43.9)	Ref
Multiple races	**124**	8 (6.5)	—	30 (24.2)	0.63 (0.47–0.86)**
Hispanic or Latina	**643**	71 (11.0)	1.28 (0.79–2.08)	263 (40.9)	0.90 (0.73–1.11)
**Poverty^¶¶^**					
Above Federal poverty level	**585**	46 (7.9)	Ref	140 (23.9)	Ref
At or below Federal poverty level	**1,008**	108 (10.7)	1.29 (0.94–1.77)	365 (36.2)	1.42 (1.25–1.62)**
Homeless past 12 months***					
No	**936**	78 (8.3)	Ref	240 (25.6)	Ref
Currently homeless	**364**	49 (13.5)	1.72 (1.24–2.39)	160 (44.0)	1.67 (1.39–2.00)**
Homeless during the past 12 months but not currently	**306**	31 (10.1)	1.30 (0.89–1.89)	113 (36.9)	1.48 (1.19–1.83)**
**Severe food insecurity past 12 months^†††^**					
Yes	**637**	103 (16.2)	2.72 (2.18–3.39)	291 (45.7)	1.87 (1.59–2.20)**
No	**968**	55 (5.7)	Ref	221 (22.8)	Ref
**Incarceration^§§§^**					
Never incarcerated	**670**	69 (10.3)	Ref	209 (31.2)	Ref
Incarcerated >12 months ago	**658**	63 (9.6)	0.95 (0.75–1.21)	183 (27.8)	0.89 (0.76–1.04)
Incarcerated past 12 months ago	**277**	26 (9.4)	0.92 (0.62–1.38)	120 (43.3)	1.29 (1.16–1.45)**
**Received money or goods in exchange for sex past 12 months**					
Yes	**549**	54 (9.8)	1.08 (0.86–1.36)	217 (39.5)	1.45 (1.25–1.69)**
No	**1,058**	104 (9.8)	Ref	295 (27.9)	Ref
**Disability status^¶¶¶^**					
Yes	**853**	92 (10.8)	1.31 (0.99–1.72)	310 (36.3)	1.41 (1.17–1.70)**
No	**747**	66 (8.8)	Ref	200 (26.8)	Ref
NHBS HIV test result****					
Negative	**902**	95 (10.5)	Ref	337 (25.5)	Ref
Positive	**659**	61 (9.3)	1.00 (0.73–1.38)	168 (37.4)	0.80 (0.69–0.94)**

**Abbreviations:** aPR = adjusted prevalence ratio; NHBS = National HIV Behavioral Surveillance; Ref = referent group.

* Atlanta, GA; Los Angeles, CA; New Orleans, LA; New York City, NY; Philadelphia, PA; San Francisco, CA; and Seattle, WA.

^†^ N = 1,608 participants. Numbers might not sum to totals because of missing data.

^§^ Row percentages.

^¶^ Models are adjusted for network size and urban area.

** Statistically significant; 95% CIs do not cross the null of 1.0.

^††^ Persons of Hispanic or Latina (Hispanic) origin might be of any race but are categorized as Hispanic; all racial groups are non-Hispanic.

^§§^ Models were not conducted for fields with sparse data.

^¶¶^ 2019 Federal poverty level thresholds were calculated on the basis of U.S. Department of Health and Human Services Federal poverty level guidelines (https://aspe.hhs.gov/topics/poverty-economic-mobility/poverty-guidelines/prior-hhs-poverty-guidelines-federal-register-references/2019-poverty-guidelines).

*** Living on the street, in a shelter, in a single room occupancy hotel, or in a car.

^†††^ Not eating for a whole day because there was not enough money for food at some point during the past 12 months.

^§§§^ Held in a detention center, jail, or prison for >24 hours.

^¶¶¶^ Serious difficulty hearing, seeing, doing cognitive tasks, walking or climbing stairs, dressing or bathing, or doing errands alone. Adjusted for age. Based on U.S. Department of Health and Human Services disability data standard (https://aspe.hhs.gov/reports/hhs-implementation-guidance-data-collection-standards-race-ethnicity-sex-primary-language-disability-0).

**** Participants with a reactive rapid NHBS HIV test result supported by a second rapid test or supplemental laboratory-based testing. Adjusted for age.

Transgender women aged <50 years were more likely to have trouble getting a job than transgender women who were aged ≥50 years. Transgender women who had income at or below the Federal poverty level, experienced homelessness, experienced severe food insecurity during the past year, had been incarcerated during the past year, had received money or goods in exchange for sex during the past year, or had a disability were more likely to have had trouble getting a job than transgender women who did not have those experiences. Transgender women who were Black or African American (Black) or multiracial were less likely to have trouble getting a job than White transgender women. (Persons of Hispanic or Latina [Hispanic] origin might be of any race but are categorized as Hispanic; all racial groups are non-Hispanic.)

Having trouble getting a job was related to health care access and use (Table 3). Among transgender women who had trouble getting a job because of being transgender, 62.4% had Medicaid only, 21.6% were uninsured, and 7.2% had private health insurance only. Transgender women who had Medicaid were 1.57 times as likely to have trouble getting a job as those with private insurance only. Although most (81.5%) participants lived in states where Medicaid explicitly covers gender-affirming care, transgender women who lived in states where Medicaid does not explicitly cover this care were twice as likely to report difficulty getting a job. Transgender women who had an unmet need for health care because of cost and never had transgender-specific health care were more likely to have trouble getting a job than those who did not. Most transgender women visited a health care provider during the past year, were currently taking hormones, or had insurance coverage for hormones; no differences were found because of high prevalence of these variables. Among transgender women who used any hormones, those who used nonprescription hormones were 1.24 times as likely to have had trouble getting a job as transgender women who did not. Transgender women who had an unmet need for gender-affirmation procedures were more likely to have trouble getting a job than those with no unmet need.

**TABLE 3:** Number and percentage of transgender women having trouble getting a job during the past 12 months, by health care access and use — National HIV Behavioral Surveillance Among Transgender Women, seven urban areas,* United States, 2019–2020^†^

Characteristic	Total no.	Trouble getting a job (n = 513)	
		No. (%)^§^	aPR^¶^ (95% CI)
**Health care access**			
**Current health insurance coverage**			
Uninsured	**270**	111 (41.1)	1.74 (1.38–2.20)**
Private insurance only	**173**	37 (21.4)	Ref
Medicaid only	**910**	320 (35.2)	1.57 (1.25–1.97)**
Medicare only	**44**	4 (9.1)	—^††^
Multiple insurance types	**143**	21 (14.7)	0.88 (0.59–1.30)
Other insurance type	**66**	19 (28.8)	1.25 (0.80–1.95)
**State Medicaid laws explicitly covered gender-affirming care, 2019^§§^**			
Yes	**1,311**	407 (31.0)	Ref
No	**297**	106 (35.7)	2.02 (1.10–3.71)**
**Usual source of health care**			
Yes	**1,325**	406 (30.6)	Ref
No	**279**	105 (37.6)	1.21 (0.98–1.48)
**Unmet need for health care because of cost past 12 months**			
Yes	**323**	170 (52.6)	1.74 (1.47–2.07)**
No	**1,285**	343 (26.7)	Ref
**Health insurance covers hormone therapy^¶¶^**			
Yes	**1,101**	323 (29.8)	0.78 (0.57–1.06)
No	**71**	26 (37.7)	Ref
Transgender-specific health care***			
Current	**1,251**	375 (30.0)	Ref
Past but not current	**143**	50 (35.0)	1.03 (0.85–1.25)
Never	**208**	84 (40.4)	1.22 (1.03–1.44)**
**Health care use**			
**Visited a health care provider past 12 months**			
Yes	**1,502**	478 (31.8)	0.94 (0.78–1.14)
No	**105**	35 (33.3)	Ref
**Unmet need for hormone therapy**			
Currently taking any hormones	**1,149**	350 (30.5)	Ref
Do not want to take hormones	**121**	41 (33.9)	1.04 (0.86–1.26)
Want to take hormones	**317**	114 (36.0)	1.12 (0.93–1.36)
**Used hormones, nonprescription^†††^**			
Yes	**246**	98 (40.2)	1.24 (1.03–1.50)**
No	**1,009**	304 (30.6)	Ref
**Unmet need for gender-affirmation procedures^§§§^**			
No unmet need	**448**	101 (22.5)	Ref
Had procedures, wants more procedures	**232**	60 (25.9)	1.16 (0.87–1.53)
Wants but has not received procedures	**840**	327 (38.9)	1.44 (1.28–1.61)**

**Abbreviations:** aPR = adjusted prevalence ratio; Ref = Referent group.

* Atlanta, GA; Los Angeles, CA; New Orleans, LA; New York City, NY; Philadelphia, PA; San Francisco, CA; and Seattle, WA.

^†^ N = 1,608 participants. Numbers might not sum to totals because of missing data.

^§^ Row percentages.

^¶^ Models are adjusted for network size, urban area, and age.

** Statistically significant; 95% CIs do not cross the null of 1.0.

^††^ Models were not conducted for fields with sparse data.

^§§^ State Medicaid coverage as of 2019 was determined by the Williams Institute’s October 2019 report (https://williamsinstitute.law.ucla.edu/wp-content/uploads/Medicaid-Gender-Care-Oct-2019.pdf).

^¶¶^ Limited to persons with health insurance.

*** Has had a provider with whom they are comfortable discussing gender-related issues.

^†††^ Limited to persons who currently use any hormones.

^§§§^ Vaginoplasty, orchiectomy, or breast augmentation.

## Discussion

Seven in 10 transgender women experienced transphobic discrimination, and one in three reported employment discrimination during the past year. Having trouble getting a job because of being transgender was associated with poor social determinants of health and lower health care access and use, including gender-affirming procedures.

The prevalence of discrimination in NHBS-Trans had certain similarities to and differences from previous studies, including the 2015 U.S. Transgender Survey (USTS) (*3*). Compared with USTS participants, NHBS-Trans participants reported similar prevalence for employment discrimination (32% NHBS-Trans versus 30% USTS); higher prevalence of bathroom discrimination (22% versus 9%), poorer treatment in businesses (39% versus 31%), verbal abuse (59% versus 12%), and physical abuse (27% versus 1%); and lower prevalence of housing discrimination (13% versus 23%) and health care discrimination (11% versus 33%). These differences might be partially explained by the sociodemographic composition of these two surveys: participants in the NHBS-Trans sample were predominantly Black or Hispanic and had lower SES, whereas participants in the USTS sample were predominantly White and had higher SES. In addition, during 2015–2019, transgender persons reported increased discrimination and minority stress because of a political climate that was increasingly hostile toward transgender persons (*20*). Finally, NHBS-Trans and USTS had differences in their questionnaires.

Employment discrimination occurs at the overlapping nexus of poverty, homelessness, incarceration, health insurance, disability, food insecurity, and survival sex work. These issues are interconnected. When economically marginalized transgender women are refused employment, this refusal cyclically contributes to economic hardships and might lead them to engage in survival sex work (*8*) and potentially incarceration, increasing their chances of facing further employment discrimination. For many persons, sex work might be their main form of employment, and employment discrimination also might occur as a part of sex work; however, that could not be examined in this analysis. In addition, although discriminating against job candidates with a disability is illegal, one third of transgender women who had a disability reported trouble getting a job. Previous studies found that transgender persons with disabilities experience high rates of employment discrimination (*21*), such as not receiving reasonable accommodations.

Employment discrimination was associated with poorer health care access, including being uninsured, having an unmet medical need because of cost, and never having transgender-specific health care. Private health insurance plans often have more provider choices and higher quality of care (*22*); therefore, employment might influence a person’s ability and opportunity to choose a gender-affirming provider, which is associated with engagement in care and improved health behaviors (*23*,*24*). In addition, having a provider with whom the person is comfortable discussing gender issues is related to pre-exposure prophylaxis use for HIV-negative transgender women (*25*,*26*) and engagement in HIV care among transgender women with HIV infection (*24*). Because transgender women who experienced employment discrimination were more likely to have no health insurance coverage or coverage through Medicaid only, improving health care staff members’ cultural competency and respect in serving transgender patients, regardless of their health insurance coverage, and increasing staff members’ representation of persons of transgender experience in health care settings is important (*27*).

The majority of transgender women in NHBS-Trans had Medicaid, which is the largest source of insurance coverage for persons with HIV infection (*28*). Four in 10 transgender women had an HIV-positive diagnosis and half reported having a disability. Therefore, the finding that Medicaid was the most common source of insurance was not unexpected. Employers also might discriminate against transgender women in part because they have low income (*29*), have an HIV-positive diagnosis (*30*), or have a disability (*21*), which is interrelated with qualifying for Medicaid.

The type of health insurance coverage that is available to transgender women is related to employment and disability status. For example, Medicaid can function as a safety net for persons experiencing sudden unemployment (*31*). Expanding Medicaid could help transgender women without health insurance qualify for Medicaid; however, Medicaid coverage of gender-affirming care varies by state (*11*,*32*). These variations can be a barrier for medically necessary health care for transgender persons with low income (*33*). In NHBS-Trans, most participants lived in states in which Medicaid programs explicitly cover gender-affirming care, with the exception of Georgia and Louisiana (*18*). This variable is likely a proxy for larger structural factors, such as negative community attitudes toward transgender persons (*34*), which can influence Medicaid policy in certain states (*35*). Furthermore, states that have not expanded Medicaid are primarily in the South, which has large numbers of Black and Hispanic residents (*36*). Historically, Medicaid policy has been shaped by structural racism, which has contributed to health inequities among Black and Hispanic persons (*36*).

Most transgender women visited a health care provider or currently use hormones; no association for these experiences was found with employment discrimination. Engagement with the health care system is usually necessary for those who desire hormones or other gender-affirming procedures; therefore, transgender women are highly motivated to seek health care and pursue hormone therapy, sometimes even at the expense of other basic needs (*37*,*38*). To achieve their transition goals, certain transgender women might even seek nonprescription hormones, which can be dangerous and unregulated (*39*,*40*), or ration prescription hormones because of cost (*41*). Improving health insurance coverage of gender-affirming care across all states could help protect transgender women from pursuing dangerous alternatives to prescription hormones. However, obtaining gender-affirming procedures without health insurance is more difficult; thus, the relation of an unmet need for gender-affirming procedures with employment discrimination is notable, which might be a structural barrier to health care access. Transgender women possibly have lower access to gender-affirming procedures in part because of employers refusing to hire them, and therefore being uninsured or inadequately insured.

## Limitations

General limitations for the NHBS-Trans are available in the overview and methodology report of this supplement (*17*). The findings in this report are subject to at least five additional limitations. First, because transgender women are hard to reach, the data might not be representative of all transgender women residing in the seven urban areas. Second, the data are self-reported and subject to recall and social desirability biases, which could underestimate results. Third, causality cannot be inferred because of the cross-sectional study design. For example, whether employment discrimination directly caused loss of health insurance or care outcomes is unknown. Fourth, whether participants are employed, how many jobs they hold, or sectors of employment where they faced discrimination is unknown. Nevertheless, transgender persons are twice as likely to be unemployed as cisgender persons (*42*). Finally, the discrimination questions were limited to transphobia and thus lack an intersectional framework. Transgender women could face discrimination because of race and ethnicity, age, weight, income, disability, and other characteristics that were not collected in the survey. Black transgender women experience unique marginalization differently from White transgender women or Black cisgender persons (*43*). This analysis indicated that Black transgender women reported less employment discrimination than White transgender women; however, this finding might be attributable to unmeasured intersectionality and not demonstrative of less discrimination. Previous studies have found that Black transgender women experience high employment discrimination (*43*,*44*); however, they are more likely to attribute discrimination to racism (*45*). Asking Black and Hispanic transgender women if they experienced discrimination solely because of being transgender likely explains some of the discrepancies between this study and other studies. Furthermore, Black and Hispanic transgender women often report mistrust in institutional systems and, therefore, might be reluctant to apply for jobs out of fear of anticipated discrimination (*8*), which could result in fewer discriminatory situations. Previous studies demonstrate that transgender persons sometimes strategically avoid certain jobs on the basis of perceptions of anticipated discrimination (*44*).

## Conclusion

Transgender women face many types of discrimination, which contribute to their economic and social marginalization. A transgender person’s ability to pursue their life goals and express their identity is compromised by lack of health insurance coverage for gender-affirming care (*33*), banning gender-affirming care for minors, and state bans that deny access to gender-affirming bathrooms (*46*). To that end, the findings from this report might be useful to guide legal, health care, and employment efforts to address threats to transgender women’s rights. Although discrimination on the basis of gender identity is illegal, employment discrimination toward transgender women still occurs; lawyers, legislators, and others can work to ensure those laws are enforced. Transgender women who have been discriminated against in the workplace can file lawsuits or complaints with the Equal Employment Opportunity Commission (*47*). Other legislative actions that have improved access to health insurance and health care include Medicaid expansion (*6*) and explicit Medicaid coverage of gender-affirming care (*48*). Employers across sectors can implement antidiscrimination trainings and policies that protect transgender women from hiring and workplace discrimination. Increased representation of transgender persons across workplace sectors might help avoid bias and build cultural competency. At an individual level, persons who are not transgender can help reduce workplace discrimination through self-education and providing social support to transgender colleagues. This analysis, which examined how employment discrimination is associated with lower health care access and use for transgender women, demonstrates the importance of transgender women working and living with dignity and without fear of unfair treatment.

## Footnotes

* 45 C.F.R. part 46.102(l)(2), 21 C.F.R. part 56; 42 U.S.C. Sect. 241(d); 5 U.S.C. Sect. 552a; 44 U.S.C. Sect. 3501 et seq.
